# Editorial: Processing of face and other animacy cues in the brain

**DOI:** 10.3389/fpsyg.2026.1799217

**Published:** 2026-03-23

**Authors:** Giorgio Vallortigara, Ichiro Fujita, Marina A. Pavlova

**Affiliations:** 1 Center for Mind/Brain Sciences, University of Trento, Rovereto, Italy; 2 Graduate School of Frontier Biosciences, The University of Osaka, Suita, Japan; 3 Research Organization of Science and Technology, Ritsumeikan University, Kusatsu, Japan; 4 Graduate School of Informatics, Kansai University, Takatsuki, Japan; 5 Department of Psychiatry and Psychotherapy, Tübingen Center for Mental Health (TüCMH), Tübingen Medical School and University Hospital, Eberhard Karls University of Tübingen, Tübingen, Germany

**Keywords:** animacy detection, autistic spectrum disorder, biological motion, face recognition, social brain, theory of mind

Over the last two decades, substantial progress has been made in our understanding of the mechanisms of animacy recognition, the ability to detect the presence of living entities in the environment (Pavlova, 2012; Lorenzi and Vallortigara, 2021). This is conceivably one of the most vital abilities that an animal should possess, as it allows proper alertness toward probable interactions, or, more specifically, prompt identification of others as valuable social companions, mates, prey, or predators. We have understood that animacy detectors operate from the very beginning after birth (Bidet-Ildei et al., 2014; Di Giorgio et al., 2017) and we have started to elucidate the underlying mechanisms of some of these “life detectors” (Buiatti et al., 2019; Kobylkov et al., 2024; Romagnano et al., 2024), mostly with respect to the selection of faces as well as face-like non-faces from similar patterns. The study of animacy is of broad interest because the brains of all vertebrates—and possibly also some invertebrates—seem to be predisposed to tell apart animate and non-animate entities. This basic, ontological ability to select animate entities has a profound impact on the development of the brain and mind.

First and foremost, animacy detection mechanisms are the bricks for building up the social brain. A growing body of evidence suggests that the lack or delayed development of animacy detectors is ultimately associated with neurodevelopmental disorders such as autism spectrum disorders (Jones and Klin, 2013; Pavlova et al., 2017; Shultz et al., 2018; Di Giorgio et al., 2016, 2021; Lorenzi et al., 2019; Galli et al., 2025). Detection of animacy also sets the stage for recognition of agency (e.g., when agents exhibit motion which is reciprocally contingent in space and time; Lemaire et al., 2022), in other words, the understanding that an animate entity may possess social characteristics such as desires, beliefs, and goals, a cognitive capacity commonly dubbed as “*theory of mind*.” Intriguingly, the distinction between animate and non-animate objects is at the foundation of intuitive dualism and essentialism (Bloom, 2004). The animate–inanimate distinction is foundational because it is the earliest cognitive division that links animacy/agency to minds and kinds to hidden essences, from which intuitive dualism and essentialism naturally emerge. Again, some fascinating evidence has been collected in recent years for attenuated dualism and essentialism in people who show reduced expression (or over-expression) of animacy and agency detectors (Berent et al., 2022; Berent and Hooley, 2025). Both under- and overexpression of animacy detection are ultimately associated with a wide range of mental disorders such as the autism, borderline personality disorder, social anxiety, and psychosis. Implication for the origins of beliefs, in particular religious beliefs have been harassed with regards to the animate/inanimate distinction (Bloom, 2004; Girotto et al., 2014; Vallortigara, 2012).

The collection of papers in the Research Topic “*Processing of Faces and Other Animacy Cues in the Brain*” (https://www.frontiersin.org/research-topics/60317/processing-of-face-and-other-animacy-cues-in-the-brain) consists of 12 contributions (with 33 authors circling around this theme; they were edited by the international and interdisciplinary team of researchers) representing the state of the art in animacy research. Most of them are critical reviews and theoretical analyses of previous work. One fascinating aspect of this Research Topic is the inclusion of papers that investigate unusual animal model systems: animacy processing is investigated in fish by Kohda et al. and in dogs by Abdai. De Agrò et al. provide a valuable introduction to largely neglected topics, namely the presence of mechanisms for the detection of animacy among invertebrates. This indicates path-breaking motion of neuroscience from a traditional primatocentric or mammalocentric attitude toward a more comprehensive and truly Darwinian comparative approach.

Given that most of the studies in this area privileged vision, it is important, as Gonan et al. do, to stress that a sense of animacy can be conveyed by other sensory modalities, such as audition.

The importance of animacy detection with respect to neurodevelopmental disorders is emphasized by the analysis of Sgadò et al., which deals with impairments in face processing in animal models and implications for our understanding of autism spectrum disorder. Complementarily, Lunghi and Di Giorgio explore how animate motion can affect visual attention in typically developing infants.

The processing of a face obviously plays a pivotal role in the collection of papers. The *vexata quaestio* of innate and learned factors in early preferences for faces is discussed by Kobylkov and Vallortigara in relation to novel data obtained through single-cell recordings in visually naive chicks. Tomonaga provides behavioral evidence for the involvement of top-down processing in face pareidolia (the perception of face-like images in random patterns) in chimpanzees (*Pan troglodytes*).

Dynamic signals from the body and faces substantially contribute to inferring social characteristics, for instance, recognizing familiar individuals. Lander and Bennetts synthesize psychological research showing that identity recognition is not solely based on static features (e.g., face structure) but also on dynamic cues such as facial movements, body actions, and vocal dynamics. This is consistent with recent neurobiological evidence that dynamic faces are processed via a cortical pathway distinct from that used for static faces in the human brain (Prabhakar et al., 2025). In this Topic, Shen et al. suggest that understanding point-light biological motion processing benefits from analysis “through the lens of animate motion processing” in the context of how the life-detection system is hardwired in the brain.

Setogawa et al. show that, alongside faces, evolutionary salient threats such as snakes are processed via a fast, subcortical pathway. While previous studies have emphasized the role of this pathway in coarse and fast processing of facial expression (Inagaki et al., 2023), they extend this framework by showing that subcortical-prefrontal circuits conserved between human and non-human primates are tuned to threatening animacy cues.

By using mobile EEG recording techniques, Soto et al. found that body posture selectively modulates early visual cortical processing but does not alter higher-level face processing, suggesting the robustness of face-sensitive neural mechanisms across bodily states.

In a nutshell, the work presented in this Research Topic nicely dovetails with and enriches the initial line of research on perceiving of animacy and inferring social signals. Yet we are far from understanding which mechanisms underlying the perception of animacy are common for animals and which of them are unique/specific for humans. Moreover, there is still a lack of cross-cultural, developmental (including healthy aging), and gender (a social construct reflecting social roles and norms)/sex (a biological construct) research. One of the most prominent assets for future studies seems to be the assessment of animacy processing in cross-disease clinical research that contributes to a fundamental understanding of animacy signals in typical development and their impairments.
